# Supplementary figures and images for: Genotypic Profile and Clinical Characteristics of CRX-Associated Retinopathy in Koreans
Source: Genes (Basel). 2023 May 8;14(5):1057. doi: 10.3390/genes14051057 (PMC10218017; doi:10.3390/genes14051057)

Mutations near or within homeobox domain

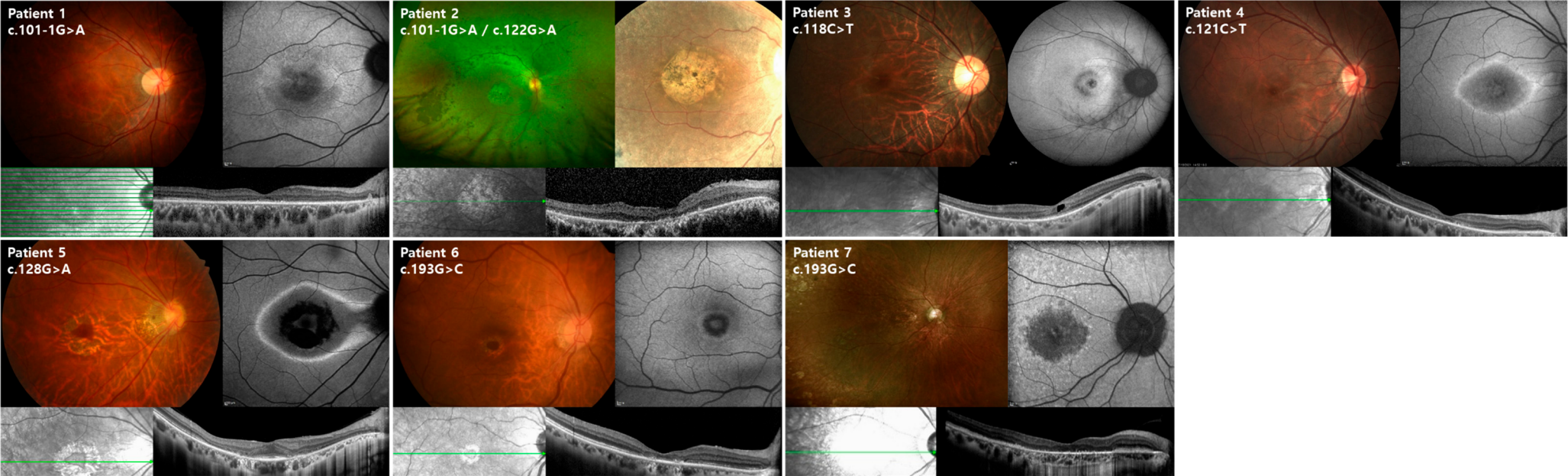

Mutations downstream of homeobox domain

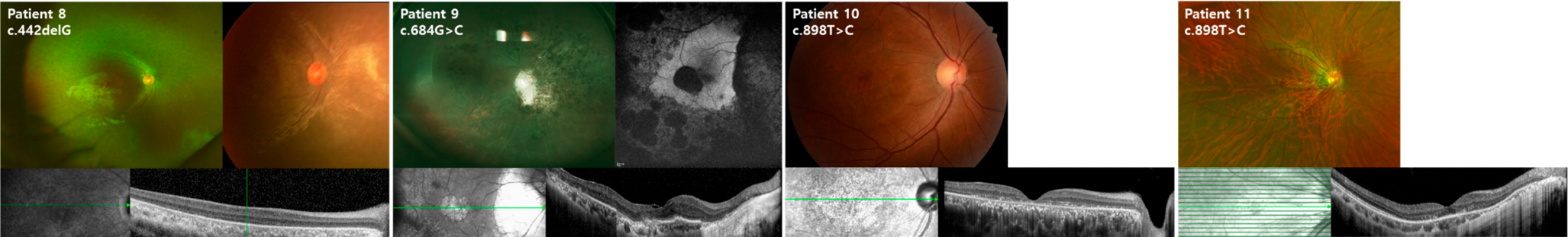

Supplement: Supplementary file 1 [file genes-14-01057-s001.zip › genes-2339007-supplementary.pdf]
